# New-Onset Insulin Allergy After COVID-19 Infection in an Insulin-Dependent Type-2 Diabetic Patient: A Rare Complication

**DOI:** 10.7759/cureus.17879

**Published:** 2021-09-10

**Authors:** Khadija Qureshi, Nauman Naeem, Javera Tariq, Maida S Chaudhry, Fajar Pasha

**Affiliations:** 1 Internal Medicine, Bucks County Kidney Specialists, Langhorne, USA; 2 Internal Medicine, Mayo Hospital Lahore, Lahore, PAK; 3 Hematology, Pakistan Institute of Medical Sciences, Islamabad, PAK; 4 Internal Medicine, DHR Health Institute For Research And Development, Edinburg, USA; 5 Internal Medicine, Holy Family Hospital, Rawalpindi, PAK

**Keywords:** diabetes type 2, corona virus disease 2019 (covid-19), insulin injection, allergy type 1, hypersensitive reaction

## Abstract

Coronavirus disease 2019 (COVID-19) is an infectious respiratory disease caused by the novel coronavirus severe acute respiratory syndrome coronavirus-2 (SARS-CoV-2). Initially, it was reported in December 2019 and became a global pandemic in March 2020, with many presentations and after-effects. We report the case of a 68-year-old female patient who presented to the emergency room with the chief complaint of a skin rash and itching all over her body, developing within a few minutes of insulin injection. The patient had tested positive for COVID-19 almost eight days ago and was self-quarantined. She was a known diabetic for the past 28 years. Her blood glucose levels were maintained within the normal range by a combination regimen of oral anti-diabetic drugs and subcutaneous humulin 70/30 (70% neutral protamine Hagedorn (NPH) insulin and 30% regular human insulin) injections. After careful examination and thorough history taking, a newly acquired insulin allergy was diagnosed in the patient, attributed to her disrupted immune system due to the recent COVID-19 infection.

## Introduction

Coronavirus disease 2019 (COVID-19) is an acute infectious respiratory disease caused by the novel coronavirus severe acute respiratory syndrome coronavirus-2 (SARS-CoV-2). Coronavirus belongs to a well-known class of enveloped and positive large single-stranded RNA viruses [1]. In late December 2019, the novel coronavirus emerged from a wholesale seafood market in Wuhan, Hubei, China. At the time of initially reported cases, bats were considered the primary hosts of this virus, and the dietary consumption of bats was deemed to cause the transmission of the novel coronavirus-2 into human cells. Within a couple of weeks, it spread not only to the other Chinese provinces but across borders, rampantly turning into a global pandemic [2]. It is mainly transmitted person-to-person through airborne droplets, while contact transmission is also possible. COVID-19 is an acute infection, mainly presenting with mild to severe pneumonia-like symptoms (headaches, fever, productive/non-productive cough, rhinorrhea, hemoptysis, shortness of breath, myalgias, arthralgias, and malaise) along with occasional gastrointestinal manifestations (diarrhea, nausea, vomiting). It is mainly diagnosed by performing a real-time polymerase chain reaction (RT-PCR) test on the nasopharyngeal swabs obtained from the patients or their blood samples [1-2]. Centers for Disease Control and Prevention (CDC) guidelines indicate an absolute need for protective measures like covering nose and mouth with masks, practicing outdoor social distancing (staying at least 6-feet apart), and implementing personal hygiene by frequent hand washing and sanitizing [3]. COVID-19 vaccines are a critical tool for controlling the ongoing global pandemic and have been actively administered worldwide, resulting in a significant reduction in the COVID-19 burden [4]. Diabetes mellitus is a group of metabolic diseases characterized by chronic hyperglycemia resulting from defects in insulin secretion, insulin action, or both. These defects are caused by the disruption in the signal transduction system at the level of insulin receptors and/or effector enzymes or genes. Diabetes mellitus is classified into type-1, type-2, gestational diabetes, and maturity-onset diabetes of the young (MODY). Type-1 diabetes mellitus (T1DM) is caused by an autoimmune loss of beta cells in the pancreas resulting in severely decreased production or complete lack of insulin. The primary mechanism of developing type-2 diabetes mellitus (T2DM) is low insulin levels and/or insulin resistance of target tissues. Type-2 diabetes has a genetic predisposition with increased free radicals, oxidative stress, and many metabolic stressors being implicated in its pathophysiology. By far, central obesity is the most common risk factor in the development of type-2 diabetes, causing insulin resistance due to excessive adipose tissue [5]. To achieve reasonable metabolic control in diabetes, a combination of lifestyle modification and pharmacological treatment is necessary. Achieving near-normal glycated hemoglobin significantly decreases the risk of macrovascular and microvascular complications. The latest advances in medicine offer various treatment options for T2DM, including both oral anti-diabetics and injectable insulin [6]. 

The human body's immune system is responsible for defense against foreign agents, microbes, and various chemical and physical agents. It has two fundamental lines of defense: innate immunity and adaptive immunity. Innate immunity is a rapid immune response with no immunologic memory. It provides protection by four types of barriers: anatomic (skin and mucous membrane), physiologic (temperature, low pH, and chemical mediators), endocytic and phagocytic, and inflammatory. Adaptive immunity is antigen-dependent and antigen-specific; it has the memory capacity, enabling the body to protect itself upon reintroducing that specific intrusive agent. The immune system can further be classified into cellular and humoral mechanisms. The cellular part includes antigen-presenting cells (APCs), antigen-specific cytotoxic T-cells, natural killer cells, dendritic cells, eosinophils, basophils, mast cells, macrophages, etc. Humoral immunity consists of antibodies, complement proteins, and antimicrobial peptides and works with its cellular counterpart, ensuring the body's defense against the invading agents [7].

However, this same immune system can lead to exaggerated immune and inflammatory responses that result in adverse outcomes known as hypersensitivity reactions. Hypersensitivity reactions are classified into four types, with type-1 being the most common cause of acute allergic reactions. Type-1 hypersensitivity reaction is caused by an acute immunoglobulin E (IgE) response against the offending agent, triggering the degranulation of mast cells and the release of histamine and other inflammatory mediators. The physical manifestations of this reaction occur mainly in the dermatological, soft tissue, ocular, nasal, and respiratory systems. It comprises an array of signs and symptoms, including skin rash, erythema, itching, wheal formation, hives, edema, rhinorrhea, lacrimation, conjunctivitis, shortness of breath, and wheezing. There are various types of antigens that the host can be exposed to, and examples include food allergies (i.e., nuts, eggs, soy, wheat, shellfish, etc.), animal source (i.e., bee, wasp, cats, insects, rats, etc.), environmental factors (i.e., dust mites, latex, pollen, mold, etc.), atopic diseases (i.e., allergic asthma, allergic rhinitis, conjunctivitis, dermatitis, etc.), transfusion reactions, and various medication-induced reactions. Several treatment options are available and depend on the severity of the reaction. These include epinephrine, antihistamines (e.g. diphenhydramine, famotidine or ranitidine, etc.), glucocorticoids, and bronchodilators (beta-agonists such as albuterol) [8]. 

## Case presentation

A 68-year-old female patient presented to the emergency room (ER) with the chief complaint of sudden onset skin rash, redness, and itching all over her body, along with shortness of breath and a generalized uncomfortable feeling. These symptoms began five minutes after administering her daily subcutaneous humulin 70/30 insulin injection (70% neutral protamine Hagedorn (NPH) insulin and 30% regular human insulin). Examination showed an erythematous, pruritic urticarial rash spread over the body and mild dyspnea, consistent with a type-1 hypersensitivity reaction. Vital monitoring showed an afebrile patient saturating 94% on room air with a blood pressure of 112/74 mmHg and a pulse rate of 87 beats per minute (bpm). Despite the dermatological findings, her vitals were stable, so she was treated with antihistamines, which led to the resolution of her signs and symptoms. Optimally intramuscular epinephrine should have been the first choice but it was not given at that time by the physician due to her vitals being normal and stable. The patient stabilized but developed a similar reaction with each subsequent insulin injection. Thus insulin was identified as the causative allergen triggering her hypersensitivity reaction. 

The patient was diagnosed with COVID-19 infection eight days ago, after which she was self-quarantined in her home. She was manifesting mild flu-like signs and symptoms at the time of diagnosis, confirmed by the positive results of the RT-PCR test performed on her nasopharyngeal swab specimen. She was retested during this ER visit for the allergic reaction, which again showed positive results for COVID-19 infection. The patient had a past medical history of T2DM, hypertension, and grade-2 diastolic dysfunction. Her daily prescribed medications include aspirin, amlodipine, nebivolol, losartan, hydrochlorothiazide, rosuvastatin, metformin, empagliflozin, humulin insulin 70/30 injections (twice daily, administered 12 hours apart), and multivitamins. The patient was compliant with her prescribed medications until she started developing severe allergic reactions to insulin a week after acquiring the COVID-19 infection. She had no previously known food or drug allergies and tolerated insulin well until this newly developed sensitivity. A disruption in the immune system due to COVID-19 infection was assumed to be the most likely cause of this hypersensitization. To avoid further allergic reactions, switching to a different insulin formulation was recommended. The patient refused treatment with a different insulin regimen despite extensive counseling. She was adamant that she has been using her regular insulin without any problems for the past 20 years, and the allergic reactions would subside with the resolution of her COVID-19 infection. She was prescribed oral antihistamines to be taken before her regular insulin injections, and also an EpiPen (epinephrine injection, USP) 0.3 mg, with the instructions to visit the ER if she develops symptoms again. 

Despite taking antihistamines, the patient occasionally developed a skin rash, erythema, and itching following insulin injections. She also experienced the side effects of antihistamines, with dizziness, malaise, and irritability being the most pronounced adverse reactions to antihistamine therapy. This conundrum led the patient to skip her insulin dosage and solely rely on oral anti-diabetics (metformin and empagliflozin). She reported total cessation of the hypersensitivity episodes after completely stopping insulin administration. The patient incorporated significant changes in her daily dietary intake to bring the preprandial and postprandial blood glucose levels into the normal diabetic range. She complained of feeling weak as a result of decreased calorie intake. Blood glucose levels were also considerably higher than her prior blood glucose readings on insulin + oral anti-diabetics regimen. Following is a chart comparing the range of her blood glucose readings on insulin + oral anti-diabetics combination therapy versus oral anti-diabetics alone (Table 1).

**Table 1:** Blood Glucose Levels On Insulin + Oral Anti-diabetics Therapy Versus Oral Anti-diabetics Alone

Time of day	Insulin + Oral Anti-diabetics	Oral Anti-diabetics Alone
Before Breakfast	115-130 mg/dL	190-200 mg/dL
2 Hours After Lunch	150-200 mg/dL	350-430 mg/dL
2 Hours After Dinner	150-190 mg/dL	400-450 mg/dL

Though the patient completely recovered from the COVID-19 infection, she continued having insulin allergies with her humulin 70/30 insulin injections. Eventually, the patient agreed to switch to different insulin formulations after about two weeks of her initial ER presentation. Due to the consistent hyperglycemic trend, she was started on twice daily preprandial short-acting lispro, to be administered before lunch and dinner. To ensure adequate basal coverage for blood glucose levels, bedtime insulin glargine was prescribed. The new insulin formulations brought the glucose levels down to her usual glycemic range without any further allergic reactions.

## Discussion

COVID-19 infection is a global pandemic that started in late December 2019, quickly crossing the international borders and posing a severe threat to the human population worldwide. It is a highly contagious and rapidly spreading infection with multiple strains, most commonly the alpha, beta, gamma and delta strains. The novel coronavirus-2 (SARS-CoV-2) has a spherical shaped core-shell with glycoprotein spikes or protein-S emerging from its surface. The surface proteins are present in whorls resembling a solar corona, thus giving the virus its name, the coronavirus. The S-proteins bind to the angiotensin-converting enzyme 2 (ACE-2) receptors on the host cells, thus allowing the viral attachment and entrance into the host cells. The ACE-2 receptors are expressed abundantly by the alveolar epithelial cells in the lower respiratory tract, thus rendering it most susceptible to COVID-19 infection [1]. Our patient also suffered the ongoing SARS-CoV-2 pandemic and became infected with the novel coronavirus-2. Her signs and symptoms of the COVID-19 disease were mild, with complete resolution of the infection after ten days. The virus did not cause any pulmonary, gastric or other commonly expected post-infectious complications in this patient. However, an unexpected consequence was an acute insulin allergy in our insulin-dependent type-2 diabetic patient, starting almost eight days after the initial infection and occurring with administering each subsequent insulin injection. These unexpected and recurrent allergic reactions made injecting her twice daily humulin 70/30 (insulin NPH and regular human insulin) dose considerably cumbersome. This allergy was thought to result from a disruption in the immune system caused by the viral particles. 

Like all other infectious viruses, coronavirus can trigger an immune response in the human body as its entry into the human cells is recognized as a foreign invasion. This infection activates a cascade of inflammatory pathways, causing a considerable immune response with a cytokine storm (CS). The innate and adaptive immunity of the host produce a variety of pro-inflammatory cytokines and activate CD4 and CD8+ T cells. These antiviral responses are essential for controlling viral replication. However, the virus could cause tissue injury by inducing excessive production of cytokines, macrophages, and granulocytes. This pro-inflammatory environment in the cells results in a CS, also called a macrophage activation syndrome (MAS) or secondary hemophagocytic lymphohistiocytosis (sHLH), thus leading to further tissue damage. The studies have shown that plasma levels of IL-1β, IL-1RA, IL-7, IL-8, IL-10, IFN-ɣ, monocyte chemoattractant peptide (MCP)-1, macrophage inflammatory protein (MIP)-1A, MIP-1B, granulocyte colony-stimulating factor (G-CSF), and tumor necrosis factor-alpha (TNF-α) are increased in patients with COVID-19. In addition, SARS-CoV-2 is responsible for immune dysregulation with the induction of aberrant cytokine and chemokine production and disrupted immune responses [9]. Insulin allergy is a rare and dreaded complication in type 1 and T2DM patients. In early documented cases of insulin allergy, hypersensitivity was developed in response to administration of animal-origin insulin; however, the introduction of human recombinant insulin in the early 1980s has led to a drastic decrease in the incidence of these cases, currently estimated at <1% to 2.4% [10]. NPH insulin, also known as isophane insulin, is an FDA-approved intermediate-acting insulin preparation created in 1946. It is the most commonly used insulin to provide basal coverage, ensuring a sustained insulin release over an extended period for blood glucose regulation [11]. Regular human insulin is a recombinant DNA insulin, synthesized mainly comprising human genetic components, and has a short duration of action. With subcutaneous use, the pharmacologic effect begins approximately 30 minutes (range: 10 to 75 minutes) after administration of doses in the 0.05 to 0.4 units/kg range. The effect is maximal at approximately 3 hours (range: 20 minutes to 7 hours) and terminates after approximately 8 hours (range: 3 to 14 hours) [12]. Humulin R-100 is a mixture of 70% NHP and 30% regular human insulin with dual short- and long-acting insulin coverage benefits. The regular human insulin part acts rapidly and lowers the postprandial glucose levels, while the NPH portion of this mixture provides basal coverage lasting up to 12-24 hours. Thus it is an exceptional alternative for the diabetic population for effective control of blood glucose levels. Side effects of insulin are mainly limited to hypoglycemia and lipodystrophy, while allergic reactions to insulin are relatively rare. If a patient is allergic to an insulin formulation, the goal is to switch to a different insulin type for lowering adverse outcomes while maintaining blood glucose levels within the normal range [11]. Though rare, there have been some documented cases of allergic reactions to insulin. The majority of the cases of insulin allergy are related to T1DM due to the autoimmune nature of the disease and an HLA association. There have been cases of patients presenting at 17 years of age with a severe allergy to short-acting insulin lispro and aspart who were subsequently treated with isophane insulin and long-acting glargine insulin [10]. Other reports show patients from age 13 to 83 years, both from type-1 and type-2 categories of diabetes mellitus, with allergies to various insulin preparations. The goal of treatment was to switch them on different formulations of insulin, thus managing their glucose levels with minimum allergic adverse effects [13]. There have been other documented cases with a case of a 60-year-old male patient with T2DM, allergic to the majority of long and short-acting insulin formulations. He was started on an insulin pump in addition to his oral hypoglycemics and achieved adequate control (glycated haemoglobin 8.3%) on 88 units of lispro per day, with little or no skin or systemic reaction [14]. The prime difference in these cases versus the hypersensitivity seen in our patient is that this patient had successfully been administering humulin 70/30 insulin injection for the past 20 years without any complication until she got infected with the novel coronavirus-2. Her immune system was uniquely dysregulated, most likely secondary to her recent COVID-19 infection, releasing inflammatory mediators which triggered an immune response against her regular insulin injection. The most likely mechanism of this newly acquired allergy to previously well-tolerated insulin seems to result from the cascade of inflammatory mediators released by activation of the T-cells. It can lead to excessive production of cytokines, interleukins, and interferons sensitizing the APCs to foreign agents (humulin insulin in this case). This drives the B-cells to form antibodies like IgE, the main culprit in producing a rapid and dramatic reaction upon re-exposure to the allergen. The various signs and symptoms of insulin allergy are the same as most of the other type-1 hypersensitivity reactions. These symptoms occur immediately or can take up to several hours to develop, persisting for variable periods. The immediate allergic reactions are comparable (but not directly proportional) to serum IgE or IgE/IgG concentrations [15]. Tendency to developing hypersensitivity towards insulin relies on its structural modifications in comparison to endogenous insulin, which would modify central tolerance for T lymphocytes. Management of such cases involves a change of insulin, but, ultimately, most individuals are set through a desensitization protocol [10]. The first step in treating moderate allergic reactions is the discontinuation of the suspected allergen and antihistamine administration. Severe allergic reactions may be treated with combinations of antihistamines in addition to epinephrine and systemic steroids. The ultimate goal is switching to a different insulin formulation and generating a proper medication regimen to appropriately maintain blood glucose levels within the normal range [16]. 

## Conclusions

COVID-19 infection is a global pandemic caused by the novel coronavirus-2 (SARS-CoV-2). This highly infectious virus can potentially trigger the immune system, disrupting its normal functioning by over-activating the inflammatory cascade. COVID-19 infection led to a newly acquired insulin allergy in our insulin-dependent type-2 diabetic patient. She had been using insulin for over 20 years and never experienced any hypersensitivity until her recent COVID-19 infection, thus evidently demonstrating that an immune disruption induced by SARS-CoV-2 was the most likely cause for her insulin intolerance. By reporting this unique case, we want the physicians to know and expect this rare complication of the ongoing COVID-19 infection. If other such patients are encountered, the physicians should suspect a possibility of a disrupted immune reaction against the viral infection and be equipped with the most recent guidelines to manage insulin or other drug allergies.
